# Expansion of the Genetic Alphabet: Unnatural Nucleobases and Their Applications

**DOI:** 10.1155/2012/718582

**Published:** 2012-12-17

**Authors:** Subhendu Sekhar Bag, Jennifer M. Heemstra, Yoshio Saito, David M. Chenoweth

**Affiliations:** ^1^Department of Chemistry, Indian Institute of Technology Guwahati, Gumahati 781039, India; ^2^Department of Chemistry and Center for Cell & Genome Science, The University of Utah, Salt Lake City, UT 84112, USA; ^3^Department of Chemical Biology and Applied Chemistry, School of Engineering, Nihon University, Koriyama, Fukushima 963-8642, Japan; ^4^Department of Chemistry, Roy and Diana Vagelos Laboratories (IAST), University of Pennsylvania, 231 South 34th Street, Philadelphia, PA 19104, USA

Nucleic acids are essential biomolecules that encode all of the information necessary for life. Specific pairing of A with T (or U) and C with G during replication, transcription, and translation is the key to effective transmission of genetic information between generations, as well as accurate conversion of genetic information into protein sequence. Given the magnitude of the tasks orchestrated by the Watson-Crick base pairing, it is striking to consider that biological systems accomplish these tasks using only four nucleobases. Realizing the powerful nature of base-pair recognition, researchers have been inspired to ask the question of whether the genetic code can be artificially expanded to generate biological systems having novel functions. It was this question that led Alex Rich in 1962 to propose the concept of orthogonal base pairing between *iso*-G and *iso*-C and inspired Professor Steven A. Benner in the late 1980s to expand the genetic alphabet from four to six letters. Benner's early research focused on the development of new base pairs having hydrogen bonding patterns orthogonal to those in the canonical Watson-Crick base pairs. In 1994, Professor Eric T. Kool opened a new functional dimension with the creation of nonhydrogen bonding unnatural nucleobase surrogates.

Expansion of the genetic alphabet has dramatically increased the functional potential of DNA, for example, by enabling site-directed oligonucleotide labeling and *in vitro *selections with oligonucleotides having increased chemical diversity. Translation of an expanded DNA alphabet into RNA is a challenging task, but one which has potential to give rise to semisynthetic organisms with increased biodiversity. This special issue highlights recent accomplishments at the interface of organic chemistry and molecular biology which hold promise to further expand the potential of nucleic acids having unnatural nucleobases. Specifically, the reports in this special issue focus on the synthesis of unnatural nucleobases and nucleic acid backbones, the exploration of their structure and duplex stabilizing ability, and the polymerase mediated replication and transcription of DNA containing unnatural nucleobases.

T. Lönnberg and a coworker report the synthesis and study of a bis(pyrazolyl)purine ribonucleoside having increased hydrophobic surface area and the ability to form complex with metal ions. The hydrogen bonding pattern of this nucleoside makes it complementary to thymine and uridine. The authors demonstrate that the bis(pyrazolyl) nucleobase is capable of forming a Pd^2+^-mediated base pair with uridine in the monomeric state. When incorporated into an oligonucleotide, the bis(pyrazolyl) nucleobase stabilizes DNA duplexes when paired with thymine, but this stabilization appears to result from increased *π*-stacking interactions rather than metal complexation. These studies open the door to applications using unnatural nucleobases to increase the binding affinity of probes and therapeutics targeted at native DNA and RNA.

Much effort has focused on the incorporation of unnatural nucleobases into native DNA and RNA, and the availability of nonnative backbones such as LNA, PNA, and GNA opens the door for further expansion of nucleic acid structure and function. C. Förster and coworkers evaluate the structural properties of “all LNA” duplexes and demonstrate that these duplexes have a relaxed helical structure unique from that of DNA and RNA. J. M. Heemstra and coworkers report an expedient synthetic route to the Fmoc-protected PNA backbone, which serves as a key intermediate in the synthesis of PNA monomers having unnatural nucleobases. Together, these studies facilitate the exploration of nucleic acids having both nonnative backbones and unnatural nucleobases, which is in turn anticipated to provide molecules having novel structural and functional properties.

The ability to use native or engineered replication and transcription machinery with nucleic acids containing unnatural nucleobases is critical to many *in vitro* and *in vivo* applications. I. Hirao and coworkers describe a two-unnatural-base-pair system capable of incorporating unnatural nucleobases into DNA via polymerase chain reaction, then into RNA via T7 transcription. Using this system, they demonstrate sequence-specific incorporation of a biotinylated nucleobase into a 260-mer RNA sequence. Generating modified RNA of this length is not feasible using purely synthetic methods; thus this work significantly improves access to site-specifically labeled RNA sequences. Additionally, P. J. Beuning and a coworker review the use of native A and Y family DNA polymerases for replication of DNA containing unnatural nucleobases. This review serves as an excellent resource for researchers seeking to utilize unnatural nucleobases and provides significant insight into how polymerases deal with both synthetic and damaged DNA nucleobases.

Collectively, these reports demonstrate the potential of unnatural nucleobases for applications in chemistry and biology and offer valuable tools for further exploring nucleic acid structural diversity. 

